# A Comparison of the Electrical Properties of Gel Polymer Electrolyte-Based Supercapacitors: A Review of Advances in Electrolyte Materials

**DOI:** 10.3390/gels10120803

**Published:** 2024-12-06

**Authors:** Ghobad Behzadi Pour, Hamed Nazarpour Fard, Leila Fekri Aval

**Affiliations:** 1 Department of Physics, East Tehran Branch, Islamic Azad University, Tehran 1866113118, Iran; 2 Department of Organic Chemistry, Faculty of Chemistry, Lorestan University, Khoram-Abad 6815144316, Iran; nazarpour.ha@lu.ac.ir; 3 Quantum Technologies Research Center, Science and Research Branch, Islamic, Azad University, Tehran 1477893855, Iran; leila2mst@yahoo.com

**Keywords:** supercapacitor, gel polymer electrolytes, ionic liquids, PVA

## Abstract

Flexible solid-state-based supercapacitors are in demand for the soft components used in electronics. The increased attention paid toward solid-state electrolytes could be due to their advantages, including no leakage, special separators, and improved safety. Gel polymer electrolytes (GPEs) are preferred in the energy storage field, likely owing to their safety, lack of leakage, and compatibility with various separators as well as their higher ionic conductivity (IC) than traditional solid electrolytes. This review covers the classification, properties, and configurations of different GPE-based supercapacitors and recent advancements that have occurred in this area of energy storage. Ionic liquid (IL)-based materials are popular GPEs for electrochemical energy storage and can be used to prepare unprecedented flexible supercapacitors due to their great IC and wide potential range. A comparative assessment of the GPEs-based supercapacitors reveals that in a majority of them, the value of specific capacitance is generally under 1000 F g^−1^, energy density reaches around 125 Wh kg^−1^, and the power density is seen to be less than 1500 W kg^−1^. The results of this research serve as an essential reference for upcoming scholars, and could significantly improve our comprehension of the efficacy of GPE-containing supercapacitors.

## 1. Introduction

The need for more efficient energy devices has led to a focus on developing electrolytes that can store and transport energy quickly, while also being stable and safe to use. The emphasis on creating flexible ionic and electronic conductive materials is growing, as these materials are crucial for various applications, including robotics, soft electronics, human interfaces, and sensors. Flexible electronics are composed of a variety of materials, encompassing both organic and inorganic elements, such as conductive polymers. Researchers are currently investigating hydrogels due to their desirable properties, including their softness, elasticity, and ability to respond to external stimuli, making them promising candidates for integration into flexible electronic devices [1]. Over the last two decades, considerable progress has been made in the field of flexible electronics, which are now employed in a wide range of applications, including sensors [2,3,4,5,6,7,8], robotics, energy storage devices, and health monitoring systems. Nanomaterials, including nanoparticles, thin films, and nanocomposites, as well as carbon nanotubes (CNTs), graphene, activated carbon (AC), and MXene, are extensively utilized in the fabrication of supercapacitor electrodes and various other applications [9,10,11,12,13,14]. Advancements in nanocomposites of Ni materials/carbon can improve conductivity, surface area, and electrochemical performance. This study outlines how Ni material-based carbon nanocomposites like Ni/graphene, Ni/CNTs, and Ni/AC affect properties like capacitance and cycling stability, serving as a resource for understanding the roles of Ni composite materials in energy storage [15]. Recent nanocomposites such as MXene with MnO_2_, W_18_O_49_, Co_3_O_4_, MoSe_2_, Fe_2_O_3_, and others have been developed as electrodes for use in supercapacitors [16].

Gel polymer electrolytes (GPEs) have emerged as a promising solution for these applications, primarily due to their excellent IC and mechanical flexibility. These materials are integral to fabricating energy storage devices, including supercapacitors, batteries, actuators, and medical sensors. The adoption of all-solid-state supercapacitors (ASSSs) utilizing GPEs is on the rise, as they provide superior ICs and enhanced safety compared to traditional liquid electrolyte-based systems. Existing GPEs encounter challenges, such as a low ionic content and potential safety risks. While the incorporation of ceramics and conducting polymers can improve IC, the weak interactions between these components may result in inefficiencies. Polyelectrolytes (PEs) are polymers that dissociate into ionized polymers and charged counter-ions in the presence of a solvent. Understanding PE behavior on charged surfaces or under confinement is crucial for various industries like biology, healthcare, energy storage, solar cell production, and paint. Due to their wide potential window and resistance to leakage, PEs are increasingly utilized as electrolytes in super-capacitors. Flexible supercapacitors are particularly useful for wearable technology, offering portability and durability. Hydrogel materials are key for creating flexible supercapacitors, providing high capacitance and power ratings [17]. The PEDOT-PVA/PEGDA double-network hydrogel supercapacitor, characterized by its flexibility and antifouling properties, represents a promising energy storage solution with significant potential for integration into wearable electronic devices [18]. A study reported the fabrication of high-performance supercapacitors using compact carbon hydrogels derived from polybenzoxazine. That study showed the potential of using polybenzoxazine-derived carbon as a sustainable and efficient alternative for energy storage devices [19]. In another study, the development and optimization of ASSSs involving carbon-based electrodes and aquivion electrolyte membranes have been investigated [20]. Recent studies indicate that co-block polymers may serve as promising materials for energy storage devices, with their structural variations under spatial confinement playing a crucial role in the design of electrodes and electrolytes [21].

**Figure 1:**
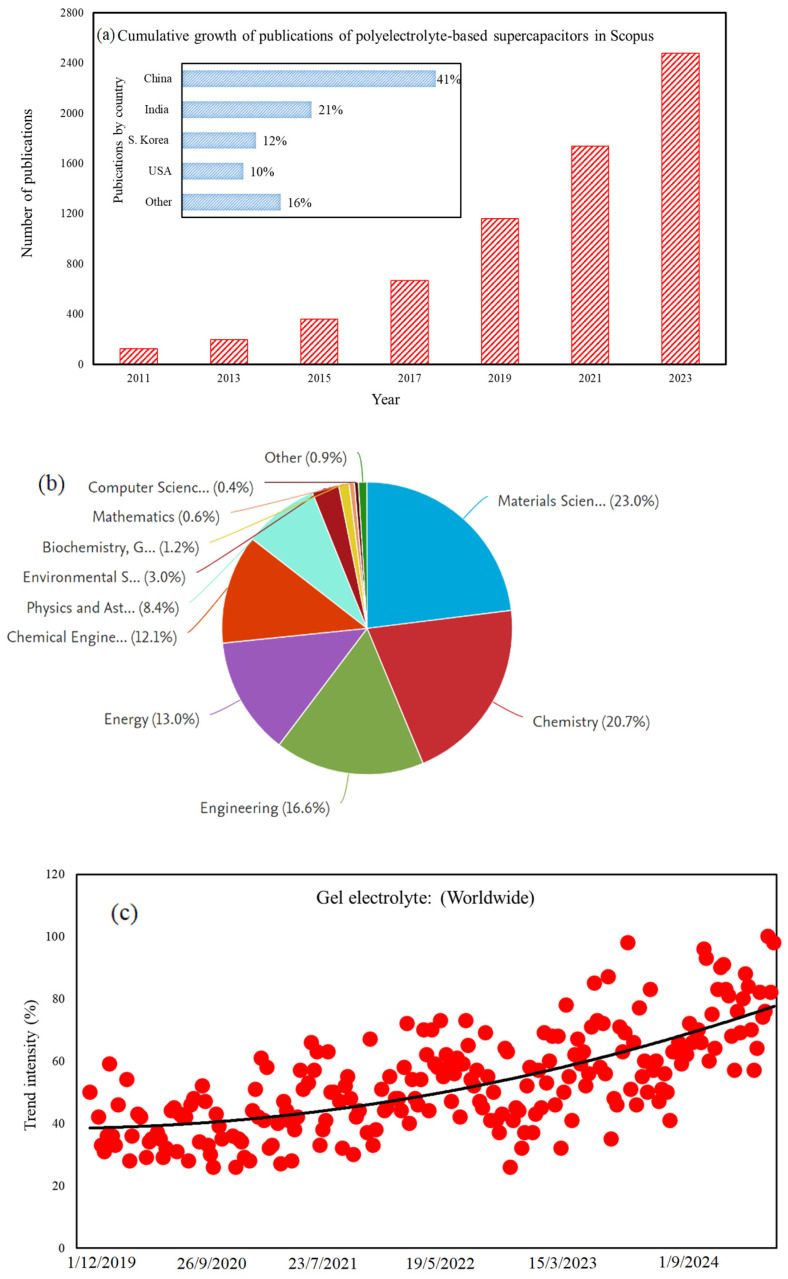
The schematic representation of (**a**) abundancy of publications, (**b**) the contributions on gel based supercapacitors in different fields, and (**c**) increasing trends toward gel electrolytes (from Google trends).

The need for more efficient energy devices has led to a focus on developing electrolytes that can store and transport energy quickly, while also being stable and safe to use. The emphasis on creating flexible ionic and electronic conductive materials is growing, as these materials are crucial for various applications, including robotics, soft electronics, and human interfaces and sensors. GPEs have emerged as a promising solution for these applications, primarily due to their excellent IC and mechanical flexibility. These materials are integral to fabricating energy storage devices, including supercapacitors, batteries, actuators, and medical sensors. The adoption of ASSSs utilizing GPEs is on the rise, as they provide superior ICs and enhanced safety compared to traditional liquid electrolyte-based systems. Existing GPEs encounter challenges, such as a low ionic content and potential safety risks. While the incorporation of ceramics and conducting polymers can improve IC, the weak interactions between these components may result in inefficiencies. Polyelectrolytes (PEs) are polymers that dissociate into ionized polymers and charged counter-ions in the presence of a solvent. Understanding PEs’ behavior on charged surfaces or under confinement is crucial for various industries like biology, healthcare, energy storage, solar cell production, and paint. Due to their wide potential window and resistance to leakage, PEs are increasingly utilized as electrolytes in supercapacitors. Figure 1 illustrates the cumulative growth in publications related to polyelectrolyte-based supercapacitors, delineates the specific subject area associated with these devices, and showcases the search trends for gel electrolytes derived from Google trends data. The global shift towards sustainable energy solutions and green technologies has spurred interest in environmentally friendly materials, such as gel polymer electrolytes, which have a potentially reduced environmental impact compared to conventional electrolytes. Rising demand for innovative energy storage solutions, advancements in materials and technology, and a focus on sustainability have contributed to the growth in articles published on Scopus and the increasing interest reflected in the Google trends results for supercapacitors based on gel polymer electrolytes.

**Figure 2:**
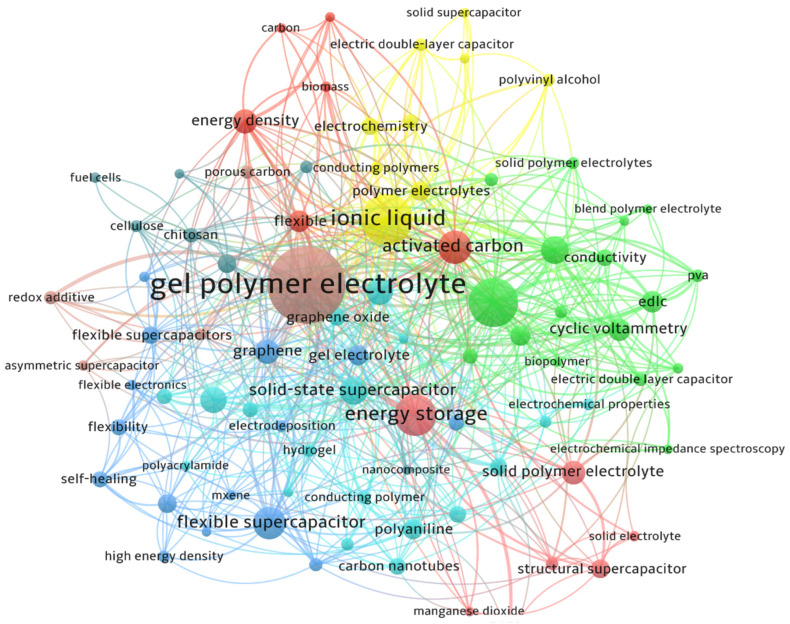
A comparative schematic of keywords extracted from reported publications on gel-based supercapacitors indexed in Scopus.

GPEs have become popular for ASSSs because of their improved mechanical properties and high IC. GPEs provide several benefits, including the prevention of leaks, the maintenance of structural integrity, and the capability to perform various functions simultaneously. Flexible and stretchable electronics, such as electronic skin and wearable electronics, have gained interest for their soft, flexible, and smart features. GPEs typically consist of a polymer matrix with various polymers like polyvinyl alcohol (PVA) and polyaniline (PANI) being used [22]. Figure 2 shows the keywords extracted from publications concerning polyelectrolyte-based supercapacitors indexed in the Scopus database. The keywords highlighted in Figure 2 include PVA, PANI, solid polymer electrolytes, blend polymer electrolytes, biopolymers, chitosan, cellulose, CNTs, MXene, graphene, active carbon (AC), and flexibility.

The comparative diagram of keywords derived from publications on gel-based supercapacitors listed in Scopus offers significant insights into the research landscape associated with this subject. This diagram reveals the most commonly utilized keywords, thereby underscoring the key areas of interest within the domain of gel-based supercapacitors. Analyzing the evolution of these keywords over time enables researchers to discern emerging trends or changes in emphasis, including advancements in materials, manufacturing methods, or potential applications. Keywords can illuminate the interrelations between gel-based supercapacitors and various other domains. A comparative examination may uncover less frequently occurring keywords, which could highlight underexplored areas in the literature that warrant further investigation by researchers. This insight can inform future research endeavors or potential funding avenues. Additionally, the keywords may reflect particular methodologies or experimental approaches that are commonly utilized in relevant studies. By integrating insights from these keywords, researchers can formulate hypotheses regarding emerging trends and pinpoint novel avenues for their investigations. Furthermore, the schematic can act as a foundational structure for conducting literature reviews or meta-analyses, enabling researchers to systematically categorize existing studies and evaluate the overall significance of diverse research fields.

PANI, as a conducting polymer, is valued for its unique properties such as its high IC, lightweight nature, and environmental stability. PANI, along with other conducting polymers like polypyrrole (PPy) and polythiophene (PTh), has extensively researched for supercapacitor applications. Despite drawbacks like low specific capacitance, combining conducting polymers with nanoparticles can overcome limitations. Electrolytes play a crucial role in energy storage systems by facilitating charge transfer between electrodes. Liquid GPEs are effective due to their composition consisting of a solvent-dissolved conducting salt and a polymer host [23]. Adding active fillers is a good method to improve the IC of polymer electrolytes by reducing polymer crystallinity. Polyvinylidene fluoride (PVDF)/polyethylene glycol (PEO) mixtures have shown significant advances in IC [24]. Shenbagavalli et al. [25] prepared solid electrolytes based on a polymer blend of PVDF/PEO containing NH_4_Br salt using a solution casting approach. They showed the supercapacitor achieved an energy density (ED) of 1.12 Wh kg^−1^ at 125 W kg^−1^. The sample exhibited the lowest level of crystallinity, demonstrating an increased IC, while the optimal potential window for the more effective conducting GPE was measured at 2 V.

Solid polymer electrolytes are crucial components in supercapacitors, although their ionic conductivities are still being improved. Hybrid/composite polymer electrolytes with ceramic reinforcements such as alumina are being explored to enhance IC and mechanical properties. A new class of GPEs has been developed through the synthesis of epoxy resin blends, which incorporate 1-ethyl-3-methyl-imidazolium bis(trifluoromethylsulfonyl)imide, lithium bis(trifluoromethylsulfonyl)imide salt, and 13 nm alumina nanoparticles, as detailed in Ref. [26]. This research indicated that adding alumina nanoparticles markedly enhances IC, which was ascribed to the interfacial interactions between the alumina and the electrolyte embedded within the polymer matrix. Conventional polymer substrates often exhibit a high crystallinity, which hinders ion migration and results in a diminished IC. A study reported that the addition of TiO_2_ nanoparticles to the solid electrolyte showed a 2.25-fold increase in the capacitance of the sodium ion supercapacitor due to a reduced crystallinity [27]. The incorporation of TiO_2_ into electrolytes serves to disrupt regions of pre-crystallinity, thereby enhancing IC and facilitating the mobility of polymer chains. This modification allows the polymer electrolyte to effectively infiltrate the electrode, resulting in improved performance characteristics for supercapacitors.

Researchers are focusing on polymeric materials for biocompatible, biodegradable, and flexible energy storage devices. Pectin, a polysaccharide found in plant cell walls and fruit peels, is being explored for use in hydrogel electrolytes for supercapacitors. Pectin-based hydrogels can conduct ions when combined with salt. A study suggested the fabrication of a thin, transparent, and flexible polymer electrolyte through a solvent casting method involving pectin, poly (ethylene glycol) (PEG), and calcium chloride (CaCl_2_), with CaCl_2_ serving as a crucial crosslinking agent [28]. That study showed that the asymmetric supercapacitor demonstrated an SC of 879 mF cm^−2^ at a current density of 15 mA cm^−2^. Sodium alginate (SA) is a naturally occurring linear biopolymer recognized for its biodegradability, affordability, and non-toxic characteristics, as well as its widespread availability from multiple sources. A robust, highly porous triple hydrogel network utilizing SA, PVA, and graphene oxide (GO) demonstrated exceptional IC, mechanical strength, self-healing capabilities, and flame retardancy. The carbon cloth-based symmetric supercapacitor constructed with this PVA/SA/GO GPE exhibited impressive performance metrics, including a specific capacitance (SC) of 633.3 mF cm^−2^ and an area ED of 84 mWh cm^−2^ [29]. With the rising energy demand, energy storage is crucial, leading to interest in biomass-based hydrogel electrolytes. Cellulose, a renewable and abundant polymer, is ideal for solid hydrogel electrolytes. Cellulose nanofibers and dissolved cellulose are common choices, but face challenges such as high costs and complex processes. The development of cellulose-based hydrogel electrolytes through green synthesis methods is crucial for addressing energy efficiency and environmental sustainability in today’s society. Another study introduced a fully eco-friendly approach utilizing an AlCl_3_/ZnCl_2_/H_2_O solvent to synthesize cellulose/polyacrylamide hydrogels characterized by intricate hierarchical structures with an area ED of 18.12 μWh cm^−2^ [30].

The production of supercapacitors via printing techniques presents a straightforward manufacturing approach, allowing for customization, enhanced material efficiency, and compatibility with a variety of substrates. Although significant research has been conducted on printable active layers, the development of printable electrolytes remains relatively underexplored. An effective printable electrolyte must possess a high ionic conductivity, an appropriate viscosity, a fine particle size, and robust chemical stability. The trend in manufacturing has increasingly favored additive techniques for the creation of diverse devices, including supercapacitors, due to benefits such as reduced waste and ease of customization. While extensive studies have focused on active layers, the formulation and evaluation of electrolytes for printed supercapacitors have not received equivalent attention, posing challenges to achieving optimal electrochemical and physical properties for reliable production. Seol et al. [31] showed a GPE exhibited a gravimetric capacitance of 176.4 F g^−1^ at a potential scan rate of 10 mV/s. Additive Manufacturing (AM) techniques enable the transformation of 2D designs into 3D functional components suitable for portable energy storage devices. Direct ink writing has emerged as a prominent AM method for the fabrication of energy storage systems, particularly supercapacitors. A newly formulated resin, incorporating conductive polymers and UV-curable substances, has demonstrated a commendable electrical conductivity and energy density in the 3D printing of supercapacitors. The development of composite material for stereolithographic printing, which combines treated PEDOT: PSS with PEGDA resin, has been optimized for both printability and electrical performance, facilitating the creation of interdigitated supercapacitors with enhanced customization for wearable electronics [32]. The integration of hydrogels into 3D-printed micro-supercapacitors reveals charge transport mechanisms and methods for improving their properties [33].

Ionic liquid (IL)-based electrolytes are promising for enhancing energy and power in supercapacitors due to their non-flammable nature and good stability. ILs show better thermal stability and safety than commercial organic electrolytes, making them suitable for various energy-related applications. Incorporating ILs into polymer matrices can widen the electrochemical window, increase IC, and enhance environmental friendliness [34]. Dicationic ILs consist of a doubly charged cation and an anion, offering more possibilities compared to monocationic ionic liquids (MILs). Molecular dynamics simulations show that DILs have less spatial heterogeneity and longer cation–anion pair lifetimes than MILs, indicating potential for improved electrolyte performances. Dicationic ILs present several advantages, including reduced toxicity, robust ion pairing, minimal volatility, and enhanced intermolecular interactions [35]. Eutectic gels utilizing sodium alginate as a natural polyelectrolyte offer a new approach to fabricating soft conductors, replacing traditional hydrogels. A double-network PVA composite eutectic gel with impressive mechanical properties and conductivity improvements was reported in Ref. [36]. That study showed that the conductivity reached 0. 30 S m^−1^, a five-fold increase, enabling the creation of a supercapacitor with wide operating capabilities and a sensor for monitoring human movement and handwriting recognition. This innovation strikes a balance between the mechanical properties and conductivity of eutectic gel, utilizing natural polyelectrolytes for high-performance ion-transport networks. In research conducted by Rathod et al. [37], a polymer electrolyte membrane composed of phosphoric acid-doped polybenzimidazole was employed to construct an ASSS, utilizing hydrous RuO_2_/carbon electrodes. It was demonstrated that this supercapacitor is capable of functioning at 150 °C to yield an SC of 421.4 F g^−1^ and an ED of 8 Wh kg^−1^ at 935.6 W kg^−1^. Yu et al. [38] developed an electrode composed of folic acid-derived nitrogen, boron, and oxygen-codoped carbon nanoparticles, which was paired with an imidazolium-based GPE for use in high-energy-density flexible supercapacitors. They achieved an ED of 32.61 Wh kg^−1^ at 357.7 W kg^−1^. In a separate investigation, symmetric supercapacitors constructed using an electrolyte of 1-ethyl-3-methylimidazolium tetrafluoroborate and electrodes made from hierarchical porous honeycomb-like carbon frameworks demonstrated an SC of 174 F g^−1^ and an ED of 74 Wh kg^−1^ at 872 W kg^−1^ [39]. This review article describes novel insights, perspectives, and comprehensive analyses of GPEs-based supercapacitors. This article specifically focuses on recent developments in GPE materials and innovative formulations. A comparison of different types of GPE supercapacitors based on their performance metrics (e.g., SC, ED, and PD) has been presented herein. This review explores new applications of GPE-based supercapacitors with integration into wearable and flexible electronics. By addressing these unique studies, the review can position itself as a valuable addition to the existing body of literature on GPEs.

## 2. Gel Electrolytes Based on the PVA Polymer

PVA is a synthetic and water-soluble polymer that is employed in various industries, including supercapacitors, papermaking, textile warp sizing, 3D printing, adhesives, and coatings. It is a colorless and odorless substance in both bead form and aqueous solutions. Table 1 presents a variety of various types of supercapacitors that are based on PVA electrolytes. Shavita et al. [40] developed a supercapacitor utilizing a composite electrode made from titanium carbide MXene and a nickel-based metal–organic framework, alongside an electrolyte composed of PVA and 1 M H_2_SO_4_. This innovative device demonstrated a voltage range of 0 to 2.0 V and achieved a PD of 331.8 W kg^−1^. In another study, a supercapacitor with a PVA/1M H_2_SO_4_ polymer gel electrolyte and an NS/Co-doped carbon electrode was reported, demonstrating an ED of 45.25 Wh kg^−1^ at 1025.64 W kg^−1^ [41]. PVA/H_2_SO_4_ gel polymer electrolyte was utilized in a solid-state symmetric supercapacitor with a sheet-like porous rGO_4_//rGO_4_ cloth as an electrode. This electrochemical system exhibited an SC of 45.86 F g^−1^ at a scan rate (SR) of 5 mV s^−1^, an ED of 1.27 Wh kg^−1^, a PD of 833.3 W kg^−1^, and an outstanding cyclic stability [42]. Co_3_S_4_/WS_2_-based composite electrodes were used to fabricate a symmetrical and all-solid-state supercapacitor using a gel electrolyte made of PVA/1 M H_2_SO_4_. The hybrid Co_3_S_4_ microspheres and WS_2_ nanorods attained an ED of 47.3 Wh kg^−1^ at 512 W kg^−1^ [43]. The hollow Co_3_S_4_ microspheres were synthesized from a highly porous precursor known as ZIF-67. To improve their energy density, these hollow Co_3_S_4_ microspheres were integrated in situ with WS_2_ nanostructures. Karuppiah et al. [44] formulated a hybrid electrolyte composed of PVA and [Co(en)_3_] Cl_3_·3H_2_O. The addition of the redox mediator into the electrolyte significantly enhanced its capacitive performance. Polymeric and gel electrolytes of PVA-H_2_SO_4_-H_3_BO_3_ (which are proton conducting) were employed in the structure of double-layered capacitors by using electrodes made of electrospinning-prepared free-standing carbon nanofibers. The electrolyte serves the roles of both the electrolyte and the separator in the supercapacitor configuration. PVA-H_2_SO_4_-H_3_BO_3_ exhibited a maximum SC and showed a great capacitance retention (CR) of 100% after 1000 cycles and yielded an excellent ED [45]. In another study, nano-TiO_2_-PVA/H_3_PO_4_ gel was prepared for use as electrolytes [46]. The initial application of various phases and particle sizes of TiO_2_ as inorganic fillers in electrolytes aimed to modify the IC by diminishing the crystallinity of the polymer matrix. The flexible supercapacitor utilizing a gel electrolyte infused with TiO_2_ demonstrated a superior energy storage performance, attributed to the optimal particle size and advantageous phases of the TiO_2_ used. Hierarchical bimetallic CoFe_2_-MOF architectures could be synthesized using the scalable solution method, as reported by Bhosale et al. [47]. The cobalt ferrite was prepared by annealing CoFe_2_-MOF in air. The supercapacitors based on these materials revealed an ED of 56.2 Wh kg^−1^ at 1091.5 W kg^−1^ with a high cyclic stability of 97.91%.

**Figure 3:**
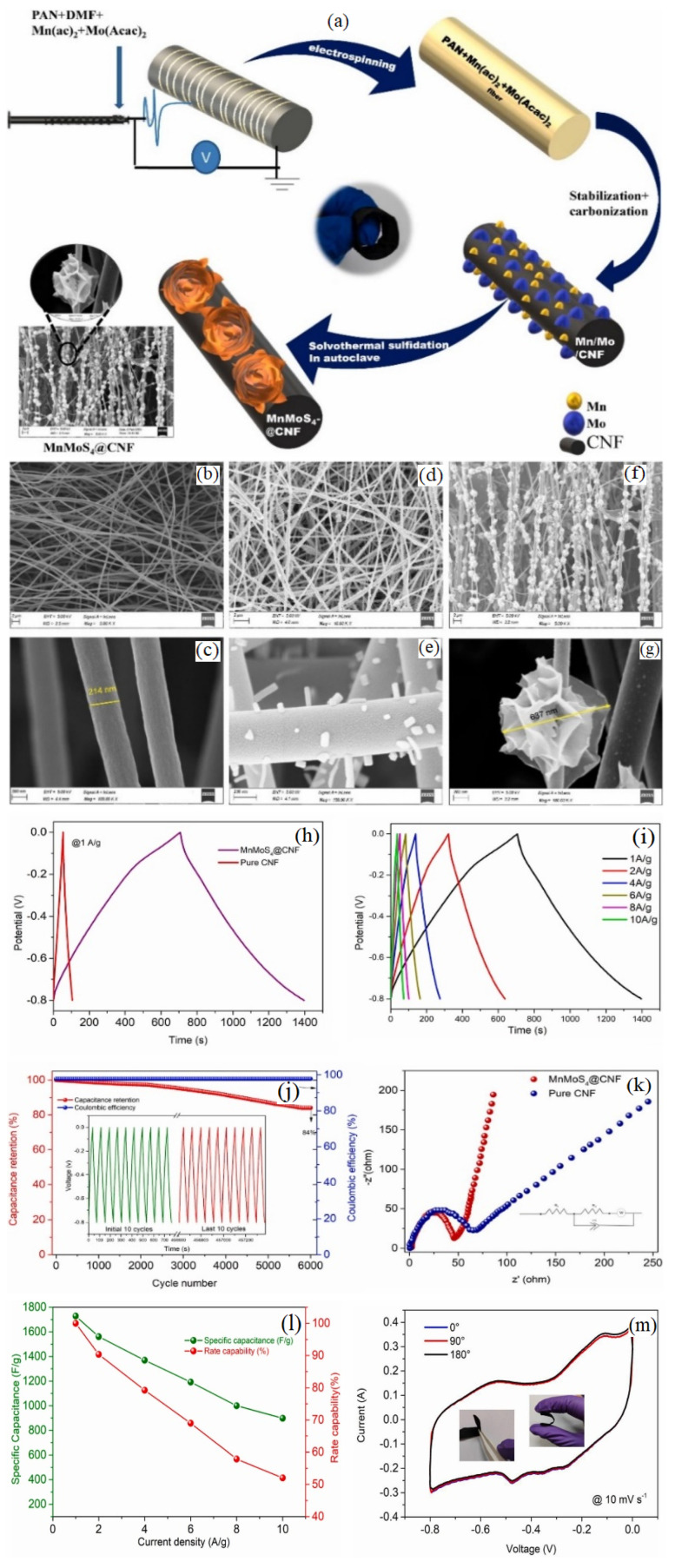
(**a**) Fabrication procedure for MnMoS_4_@CNF, and SEM images of (**b**,**c**) CNF, (**d**,**e**) MnMo@CNF, (**f**,**g**) MnMoS_4_-@CNF, and (**h**–**m**) electrochemical characteristics of the supercapacitor [48].

Self-standing core–shell manganese molybdenum sulfide/carbon nanofiber (MnMoS4@CNF) hybrid mats and PVA/KOH-based solid/gel polymeric electrolytes were developed for flexible supercapacitor uses [48]. The mats showed an SC of 1727.9 F g^−1^ at 1 A g^−1^ and 84% CR at 10 A g^−1^ after 6000 cycles. The mats used in an asymmetry with a solid supercapacitor resulted in an ED of 73.7 Wh kg^−1^ at 800 W kg^−1^. Figure 3 illustrates the process of fabricating MnMoS_4_@CNF and also the electrochemical characteristics of the prepared supercapacitor. The supercapacitor was tested at 30 mVs^−1^ in two electrode configurations, showing a stable pseudo-rectangular curve up to 1.6 V. A maximum voltage of 1.6 V was chosen for the operation. CV profiles at different scan rates within this window showed well-defined oxidation and reduction peaks with symmetrical features, indicating excellent electrochemical stability and capacitance properties. The electrochemical performance of a solid-state supercapacitor was evaluated using a galvanostatic charge–discharge (GCD) curve from a current density of 1 to 10 A g^−1^. The curve was symmetrical, showing that capacitance mainly came from faradic redox reactions. The redox peaks observed during cathodic/anodic sweeps indicated the pseudocapacitive behavior of MnMoS_4_@CNF. The accumulation of charge in aqueous electrolytes by metal sulfides generally occurs through a transformation of the metal sulfides into sulfo-hydroxide species, which subsequently convert into sulfoxide [48]:

(1) $$\left(Mn\right)S+{OH}^{-}↔MnSOH+e^{-}$$

(2) $$MnSOH+{OH}^{-}↔MnSO+H_{2}O+e^{-}$$

Aziz et al. prepared silica-doped and porous graphitic carbon (marsh clay (peat soil)-derived silica) as an electrode and PVA/KOH GPE for use as innovative components of the symmetric and ASSSs. The developed supercapacitor device delivered an SC of 160 F g^−1^ and an ED of 18 Wh kg^−1^ at 312 W kg^−1^ [49].

**Figure 4:**
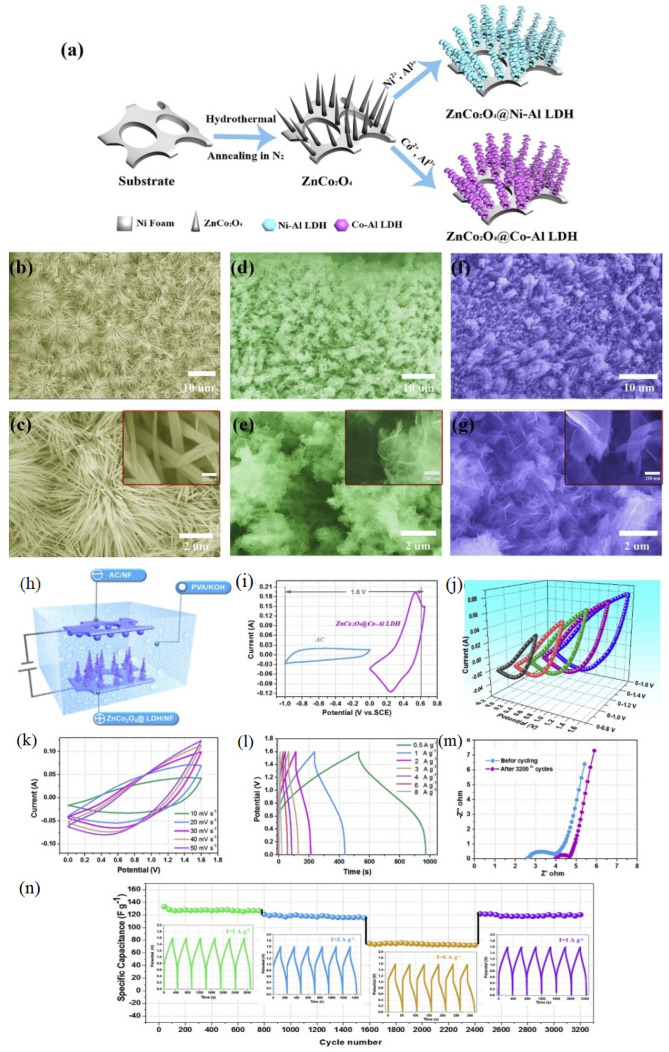
(**a**) The fabrication methodology for ZnCo_2_O_4_@LDH, SEM images of (**b**,**c**) ZnCo_2_O_4_ nanowires, (**d**,**e**) ZnCo_2_O_4_@Ni–Al LDH (**f**,**g**) ZnCo_2_O_4_@Co–Al LDH, and (**h**–**n**) electrochemical properties of the supercapacitor [51].

Another study employed porous MnMoO_4_/carbon nanofiber (CNF) as an electrode along with a polymeric electrolyte of PVA/KOH. The supercapacitor with MnMoO_4_@CNF//AC electrodes showed an ED of 37.46 Wh kg^−1^ at 278 W kg^−1^ [50]. Bai et al. [51] reported a supercapacitor based on polymer-based PVA/KOH electrolyte. In this device, ZnCo_2_O_4_ nanowires produced by using two types of typical layered double hydroxides (ZnCo_2_O_4_/Co–Al LDH and ZnCo_2_O_4_/Ni-Al LDH) were utilized as the electrode. This asymmetric kind of supercapacitor (ZnCo_2_O_4_@Co–Al LDH//AC) showed an ED of 6200 W kg^−1^ at 50.1 Wh kg^−1^. Figure 4 shows the synthesis procedure for ZnCo_2_O_4_@LDH, as well as the electrochemical properties exhibited by the supercapacitor. Comparative CV curves were used to confirm the voltage window of the devices. The results showed that the ZnCo_2_O_4_@Co–Al LDH//AC supercapacitor exhibited an excellent rate capability and stability, with a specific capacitance of 139.2 F g^−1^ at various current densities. The core–shell structure of the electrode played a significant role in providing an active surface for faradic reactions and facilitating rapid electron transport. Long-term cycling tests showed that the ZnCo_2_O_4_@Co–Al LDH//AC maintained 90.5% of its initial specific capacitance after 3200 cycles, demonstrating a superior stability and rate capability. Electrochemical impedance spectroscopy results confirmed the enhanced conductivity and reduced ion diffusion resistance of the device after cycling, further supporting the superior performance of the core–shell electrodes. The distinct redox peaks originating from the faradaic reaction can be represented by the following equations [51]:

(3) $$Zn{Co}_{2}O_{4}+H_{2}O+{OH}^{-}↔2CoOOH+ZnOOH+e^{-}$$

(4) $$CoOOH+{OH}^{-}↔CoO_{2}+H_{2}O+e^{-}$$

Cevik et al. [52] synthesized a sulfonated derivative of hollow silica spheres (HSS-S). The hollow spheres have the potential to be impregnated with acid and then blended with PVA at varying weight percentages to produce nanocomposites of GPEs. The supercapacitor device (symmetrical type) prepared from the electrolytes and an AC electrode revealed an SC of 52 F g^−1^ with a remarkable cyclic stability [52]. In another study, an alkaline GPE comprising PVA/KOH/urea/LiClO_4_ was utilized in a Co oxide/Mn oxide wearable and asymmetric supercapacitor. The experimental protocol involved selecting a deep eutectic solvent, urea/LiClO_4_, as the redox additive and utilizing a wide operating voltage range. Additionally, an alkaline gel comprising PVA-KOH was employed as a plasticizer to enhance the redox performance of the electrochemical system. The device with PVA/KOH/urea/LiClO_4_ as the gel type for electrolyte can operate with an output voltage of 2.2 V and render an ED of 125 Wh kg^−1^, an SC of 186 F g^−1^ at 2 A g^−1^, and 89.5% CR after 6000 cycles [53].

Another study introduced a solid supercapacitor based on GPE with AC electrodes originating from coconut shells. The GPE was a composite of PVA/KOH/hydroquinone obtained through a solution casting technique and showed a high IC and flexibility. The supercapacitor demonstrates an SC of 326.53 F g^−1^ with 84.2% CR after 1000 cycles [54]. Gunday et al. [55] reported flexible and high-performance supercapacitors with AC electrodes. The GPE was prepared by blending a ternary system of PVA/glycerol/boric acid hydrogel doped with LiNO_3_. The synthesized GPE retained a great IC and excellent flexibility in a broad temperature range. Also, the performance assessment of the supercapacitor devices at low and high temperatures confirmed their high stability. Moreover, the supercapacitor exhibited an SC of 396 F g^−1^ with an ED of 27.41 Wh kg^−1^ at 118 W kg^−1^ [55]. A study examined the impact of GPE with a PVA/PEO blend (90/10, wt%) and various Na salt mixtures on the electrochemical traits of ASSSs. Carbon black and graphene nanoplatelets were utilized as active electrodes. The results exhibited that a 30% content of Na salt mixture produced the highest SC of 93.768 F g^−1^ [56]. A novel graft copolymer based on poly (vinyl alcohol-co-acrylonitrile) and polyethylene glycol monomethyl ether (PEGME) was synthesized and employed to create GPEs by doping with LiBF_4_. An electrochemical capacitor device containing the GPE sample showed a high conductivity, with an SC of 105 F g^−1^ and an ED of 14.8 Wh kg^−1^ [57].

## 3. Gel Electrolytes Based on Ionic Liquids (ILs)

ILs are nonvolatile chemicals with unique behaviors that can replace volatile organic compounds in various industrial uses. A wide range of ionic liquids can be synthesized by combining different cations and anions. In other words, they are organic salts that remain liquid at temperatures below 100 °C. They have low vapor pressure, are nonflammable, and serve as eco-friendly substitutes for traditional solvents. Functionalized ionic liquids can also be developed by adding specific chemical groups to the IL structure for task-specific applications. ILs’ properties can be adjusted by changing the cation and anion components and/or their chemical structure. ILs have proven to be versatile for use as solvents or electrolytes, making them popular in renewable energy storage [58]. A comparison of different supercapacitors based on IL-based electrolytes is shown in Table 2. Hierarchical porous membranes with different pore contents at various length scales are novel materials that are growing in number and are capable of utilizing energy storage, chemical sensing, catalyst support, and water purification. The high surface area of these membrane materials can be used for balancing rapid ion transportations to enhance the IC of energy storage devices. Amphiphilic block copolymers (BCs) are key building blocks for porous membranes due to their self-assembled nanodomains. Within strategies to create BC-based electrolytes, ILs can be used to improve both mechanical properties and IC of these versatile materials. Many studies have shown that IL-containing BCs can significantly increase IC values compared to homopolymer-based electrolytes. Handayani et al. [59] designed hierarchically porous membranes based on a silica network-derived poly(styrene)-b-poly(2-vinylpyridine) copolymer with an IL electrolyte. This synthesis was achieved through the application of nonsolvent-induced phase separation alongside nonhydrolytic sol–gel techniques, which integrate a network of silica nanoparticles, resulting in the formation of porous structures at both nano- and micro-scale dimensions. The supercapacitor prepared from these materials shows an SC of ∼90 F g^−1^ at 0.2 A g^−1^ and a CR of 76% after 1000 cycles [59].

ILs are being considered as an alternative to traditional components in electrolytes due to their unique features, e.g., chemical stability and non-volatility, as well as the high capacitance and energy that appear within IL–GPE-based supercapacitors. Lee et al. [60] optimized the electrochemical behavior of ASSSs by using different kinds of IL-based GPEs. The GPE PVA/KI/ethylene carbonate/1-ethyl-3-methylimidazolium tetrafluoroborate was utilized in a supercapacitor configuration leading to an ED of 180.67 Wh kg^−1^ at 375 W kg^−1^ [60]. A study reported a GPE film with a good mechanical strength, high IC, and strong adhesive characteristics, based on a microphase-separated and partially fluorinated comb copolymer consisting of amphiphilic crystalline poly(ethylene glycol) behenyl ether methacrylate and superhydrophobic poly(2,2,2-trifluoroethyl methacrylate). A GPE film with an IL exhibited a potential window of 2.2 V and an SC of 37.3 F g^−1^ [61]. A new highly conducting IL-doped biopolymer electrolyte (ILBPE) has also been introduced [62].

Here, cornstarch as a host and a low-viscosity IL of 1-ethyl 3-methylimidazolium thiocyanate as a dopant were utilized for preparing an EDLC. An IC value of 2.6 × 10^−4^ S cm^−1^ was exhibited by this product and the ILBPE-based supercapacitor exhibited an ED of 23.13 Wh kg^−1^ at 3600 W kg^−1^ [62]. Melamine foam could be used as a convenient template to create hierarchical, high surface area, and porous carbon for electrode usage. This material was used as an electrode and 1-ethyl-3-methylimidazolium tetrafluoroborate (EMIMBF_4_) as an electrolyte in a supercapacitor. The optimized electrode reached an SC of 217 F g^−1^ at 0.1 A g^−1^ and CR of 89% after 10,000 cycles [63].

The electrochemical performance of AC electrodes has been assessed by fabricating a symmetric configuration of an electric-double layer capacitor with aqueous liquid electrolyte (1 M Na_2_SO_4_) and quasi-solid-state and IL-incorporated GPEs. The self-supporting film of an ILGPE containing 1-ethyl-3-methyl imidazolium bis(trifluoromethyl sulfonyl)imide (EMITFSI) on a copolymer of (PVDF-hexafluoropropylene) demonstrated promise as an electrolyte suitable for EDLCs. The ILGPE-based quasi-solid-state EDLC offered an SC of 180 F g^−1^. The capability of hexagonal boron nitride (hBN) as a filler in various polymer applications and its benefits for the electronics industry can be attributed to its high thermal conductivity, hydrophobicity, thermal stability, low electronic conductivity, and lubricant properties [64]. A study carried out by Gunday et al. exhibited a maximum IC value of 2.5 × 10^−4^ at 100 °C for a polymer electrolyte comprising 1-Ethyl-3-methyl-imidazolium tetrafluoroborate (IL), sulfonated polysulfone (SPSU), and hBN. The produced membranes have promising thermal and dimensional stability and a higher conductivity compared to the parent materials. A symmetrical cell of 0.2 IL/SPSU/%5 hBN/sample yielded an SC of 90.4 F g^−1^ at 1 A g^−1^ [65]. SPSU produced from sulfonated polysulfone shows an enhanced mechanical strength and stability, suitable for energy devices like supercapacitors. SiO_2_ (silica) is a conventional natural material with various non-crystalline and crystalline forms. Quartz is abundantly utilized in silicon production, ceramics, and glass. Both synthetic and natural silica particles can be employed to elevate material characteristics. Incorporating metal oxide particles into polymers can improve their conductivity, water retention, and strength [65]. Another study introduced a novel polymer electrolyte containing IL (1-Ethyl-3-methyl-imidazolium tetrafluoroborate as a softening agent), nano-SiO_2_, and SPSU [66].

**Figure 5:**
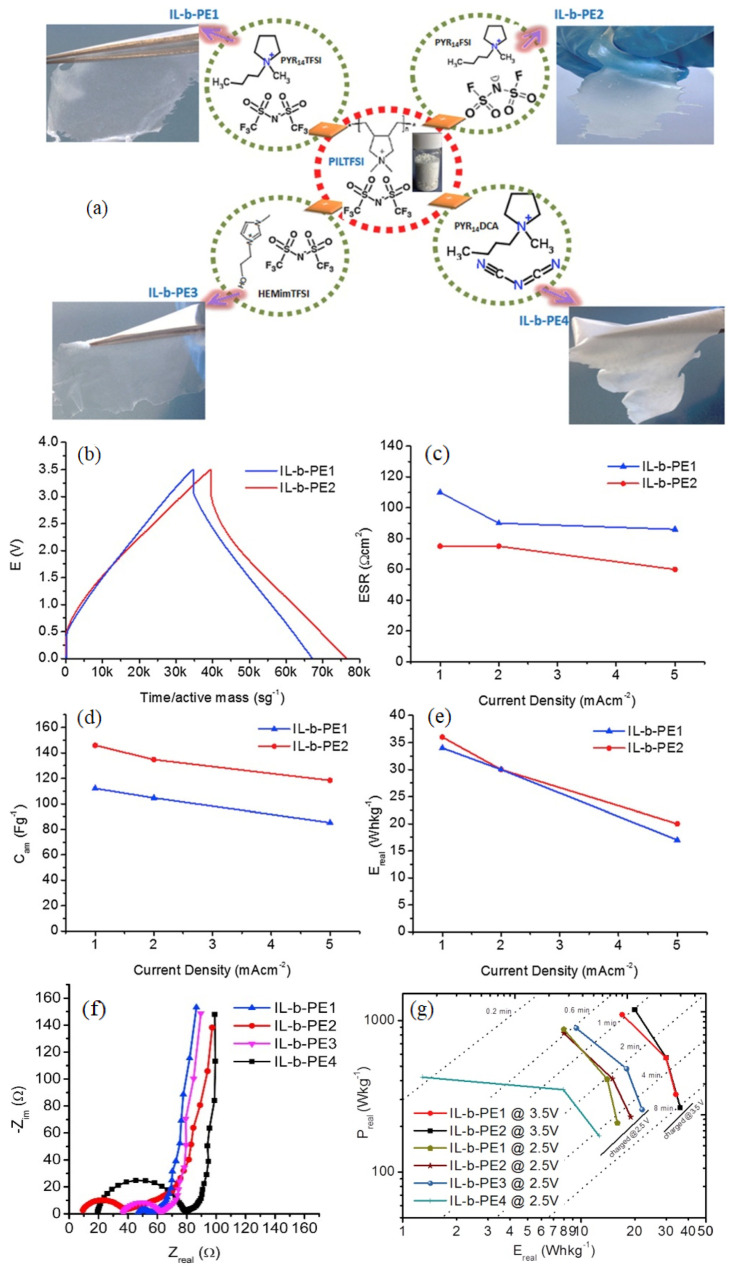
(**a**) Chemical structure of different IL-b-PE electrolytes, (**b**–**e**) electrical characterization of supercapacitors, (**f**) Nyquist plot, and (**g**) Ragone plot of the supercapacitors [68].

The prepared nano-GPEs demonstrated lower glass transition temperatures and the high thermal stability required for supercapacitor application. The highest IC values were obtained for the SPSU/0.2IL/%3SiO_2_ and SPSU/0.1IL samples with the values 3.2 × 10^−3^ and 3.1 × 10^−4^ S cm^−1^ at 100 °C, respectively. Symmetrical cell formation based on Al/C/SPSU/0.2IL//%3SiO_2_/C/Al showed an SC of 134.1 F g^−1^. The GPE composed of the IL sample (1-butyl-3-methylimidazolium chloride), Li_2_SO_4_, and PVA demonstrated a great IC of 37 mS cm^−1^. The supercapacitor fabricated with the electrolyte and AC electrodes showed a CR of 90% even after 3000 cycles and an ED of 10.6 Wh kg^−1^ at 3400 W kg^−1^ [67]. Polymeric ILs offer a new approach to designing IL-based GPEs with enhanced properties. Four ILs-based GPEs were prepared by blending a polymeric IL, poly(diallyl dimethyl ammonium) bis(trifluoro methane sulfonyl)imide, with four different ILs: 1-(2-hydroxy ethyl)-3-methylimidazolium bis(trifluoromethyl sulfonyl)imide (IL-b-PE3), 1-butyl-1-methylpyrrolidinium bis(trifluoro methane sulfonyl)imide (IL-b-PE1), 1-Butyl-1-methylpyrrolidinium dicyanamide, (IL-b-PE4), and 1-butyl-1-methylpyrrolidinium bis(fluorosulfonyl)imide (IL-b-PE2). The physicochemical properties of ILGPEs, such as IC, and electrochemical/thermal stability, were found to be dependent on the IL’s features. The supercapacitor with IL-b-PE2 electrolyte exhibited an ED of 36 Wh kg^−1^ at1170 W kg^−1^. Figure 5 reveals the chemical structure of different IL-b-PE electrolytes and the electrical characterization of the prepared GPE supercapacitors [68]. The CD profiles of solid-state supercapacitors assembled with different ILs show typical triangular and linear responses, indicating electric double-layer behavior without pseudo-capacitance or redox reactions.

Equivalent series resistance (ESR) values reveal that an SC with IL-b-PE2 has lower ESR values due to its higher conductivity. The SC of the supercapacitor increases by 15–20% when charged up to 3.5 V. Charging at 2.5 V shows varying performances due to the different properties of the ILs. Energy and power increase with higher operating voltages. The diagonal dotted lines in the chart show constant discharge rates and relative charge time for the supercapacitors. The Nyquist plot for solid-state supercapacitors indicates a capacitive behavior with a vertical line along the imaginary axis at low frequencies. The ESR can be estimated by extrapolating this line to the *x*-axis, including bulk electrolyte resistance, electrode intrinsic resistance, and interface resistance. Differences in the plot display varying electrolyte bulk resistances and ESR values at high frequencies. The pseudocapacitor based on PPy+PIL2/PIL1/PPy+PIL2 electrolyte showed an SC of 1.39 Wh kg^−1^ at 286 W kg^−1^ [69]. In another study, an epoxy-based solid-state polymer electrolyte (SSPE) containing ionic liquid, oligoether, and Li salt was found to be a promising candidate. In another study, a microscopic, fast, ion-diffusing channel was incorporated into the macroscopic cross-linked matrix through polymerization-induced microphase separation [70]. The IC, dielectric properties, and mechanical characteristics of the materials can be precisely adjusted by modifying the concentration of lithium salt. This results in a change in the relative interactions between the insulating and conducting phases, leading to the formation of various morphologies. The optimized SSPE assembled with AC electrodes exhibited an elongation at a break of 153%, an IC of ~10^−3^ S cm^−1^ at 25 °C, and achieved an SC of 178 F g^−1^. The symmetric supercapacitor composed of an N-doped AC electrode with 2 wt% along with an IL electrolyte delivered an ED of 8.6 Wh kg^−1^ and a PD of 38.9 W kg^−1^ [71]. Mun et al. [72] introduced an improved-performance GPE comprising a poly (isobornyl methacrylate)-co-poly (ethylene glycol) methyl ether methacrylate amphiphilic comb copolymer. The GPE showed a bicontinuous and worm-like nanostructure with an IC of 4.35 mS cm^−1^v [72].

## 4. GPE-Based Supercapacitors

GPEs like alkaline GPE membranes have attracted much attention for their high safety and good processability, but their mechanical properties can be rich in challenges. Among GPE matrices, PVA is commonly considered the favored choice despite its drawbacks like low elongation and poor water retention. To address this, high-strength hydrogel structures could be used to improve the mechanical properties of alkaline GPE membranes, with double network structures being particularly effective. Table 3 presents a comparison of different supercapacitors based on GPEs. Yang et al. [73] developed a GPE membrane based on PVA and kappa-carrageenan. The fabricated GPE-based supercapacitor showed an SC of 741 F g^−1^ and an ED of 18.3 Wh kg^−1^ at 800 W kg^−1^. Hydroxide Ni(OH)_2_ is a promising metal hydroxide for use in supercapacitors due to its high theoretical capacity, stability, and low cost despite its challenging poor electrical conductivity. Compositing Ni(OH)_2_ with graphene to form a composite electrode can obviate this issue. A hydrothermal method has been utilized to synthesize a Ni(OH)_2_-rGO composite, which shows a high electrochemical performance in alkaline electrolytes [74]. While these kinds of electrodes have been successfully employed in supercapacitor systems, their usage along with aqueous electrolytes has led to some limitations. Replacing liquid electrolytes with solid-state GPEs, like Li ion-based GPEs, could overcome these restrictions to reach an enhanced performance. The GPE-based system showed battery-like behavior at an operable potential of 3 V and exhibited an SC of 6.7 F g^−1^. By lowering the potential to 2 V, the supercapacitor demonstrated over 15,000 cycles with a CR of 95%. The altered performance was attributed to the material’s capacitive behavior and a faradaic reaction. The interaction between the GO solution and nickel foam during the hydrothermal process could be described as follows [74]:

(5) $$Ni+GO+O_{2}↔Ni{\left(OH\right)}_{2}+RGO+H_{2}O$$

The reduction associated with the conversion of Ni(OH)_2_ to Ni, lithium intercalation into the graphite layers, and the oxidation process can be defined using the following chemical reactions [74]:

(6) $$Ni{\left(OH\right)}_{2}+2{Li}^{+}+2e^{-}↔Ni+2LiOH$$

(7) $$x{Li}^{+}+6C+xe^{-}↔{Li}_{x}C_{6}$$

(8) $${Ni}^{2+}+2e^{-}↔Ni$$

GPEs based on (poly(VA-co-acrylonitrile))/PEO were produced and doped by LiBF_4_/1-ethyl-3-methylimidazolium (IL). In the dry state, the GPEs exhibited a conductivity of 2 × 10^−4^ S cm^−1^ at RT and 7 × 10^−3^ S cm^−1^ at 100 °C. The symmetrical cell yielded an ED of 61 Wh kg^−1^ at a PD of 500 W kg^−1^ and an SC of 80 F g^−1^ at 1 A g^−1^ [75]. A GPE from α-cellulose with excellent biodegradability, mechanical properties, and thermal stability was prepared in combination with hierarchical porous carbon electrodes, showing an SC of 133 F g^−1^. Interestingly, the GPE showed a favorable natural degradation rate under aerobic sludge conditions. Therefore, this fabricated EDLC is expected to be employed as a sustainable and green electronic device with environmental friendliness and amiable economic availability characteristics [76]. An electrolyte based on dextran (Dex)/hydroxyethyl cellulose blended with NH_4_Br achieved an SC of 31.7 F g^−1^ [77]. Novel redox-activated GPEs based on nickel nitrate Ni(NO_3_)_2_ and poly(vinylphosphonic acid) (PVPA) were prepared. Flexible supercapacitors assembled with PVPA/Ni_X_ hydrogels and AC electrodes produced high SCs with a 30-times-improved capacitance compared with the Ni-free system, as well as a high ED [78]. In another study, a GPE was synthesized using to ammonium molybdate-incorporated PVPA, and supercapacitors including AC electrodes were fabricated using PVPA/Mo with various weight fractions of Mo. All the electrolytes showed excellent stretching and bending properties. Surprisingly, the SC of the device was enhanced by at least 50 compared to the parent sample after incorporating Mo as a mediator. Also, no oxidation–reduction peak appeared in the stable PVPA hydrogel structure. The CV curve of pure Mo showed two redox peaks that are described as follows [79]:

(9) $$H_{2}Mo\left(IV\right)O_{3}+H_{2}O\to H_{2}Mo\left(VI\right)O_{4}+2e^{-}+2H^{+}$$

(10) $$HMo\left(V\right)O_{3}+H_{2}O\to H_{2}Mo\left(VI\right)O_{4}+e^{-}+H^{+}$$

A GPE based on poly(2-acrylamido-2-methyl-1-propanesulfonic acid) (PAMPS) was used to construct supercapacitors including AC electrode and nanocomposites electrodes of AC/nano-SiO_2_ and AC/multiwalled carbon nanotubes at various weight fractions. An SC of 315 F g^−1^ at 0.5 A g^−1^ was observed for the symmetrical device composed of AC electrodes, and the ED of this device was 55.5 Wh kg^−1^ at a PD of 690 W kg^−1^ [80]. GPEs including ammonium molybdate [(NH_4_)_2_MoO_4_] (Mo) and poly (2-acrylamido-2-methyl-1-propanesulfonic acid) (PAMPS) were prepared in different compositions. The system containing AC as the electrode and PAMPS/Mo_3_ as the electrolyte produced an SC of 530 F g^−1^ with a 30% enhancement in capacitance in comparison to the Mo-free supercapacitor. The ED of the device was 73 Wh kg^−1^ with a PD of 580 W kg^−1^ [81]. Wang et al. utilized the digital light projection additive manufacturing protocol for the formation of GPEs for flexible supercapacitors based on crosslinked poly(acrylic acid-co-vinyl imidazole) through an equilibration with lithium chloride in deionized water, ethylene glycol, and ethylene glycol/deionized water mixtures. The supercapacitor exhibited an SC of 60 F g^−1^ [82]. Chitosan-based hydrogels (particularly carboxylate chitosan) have shown a capability to serve as environmentally friendly electrolytes. Carboxylate chitosan, with its pH sensitivity, biodegradability, and biocompatibility, is suirtable for a wide range of applications but has not been extensively studied as an electrolyte material for supercapacitors. The fabrication of eco-friendly, transparent, and flexible GPE films based on biodegradable carboxylated chitosan via the phase separation of carboxylated chitosan in hydrochloric acid has been reported in Ref. [83].

**Figure 6:**
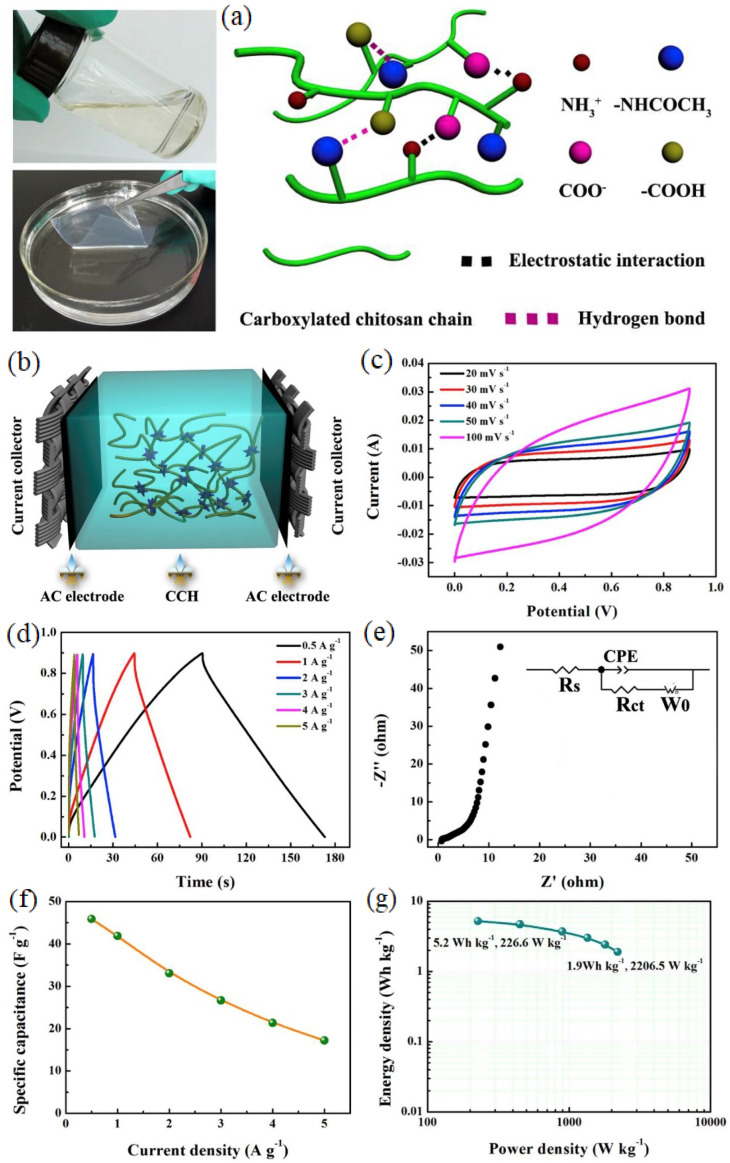
(**a**) Carboxylate chitosan solution; (**b**) schematic of assembled EDLC device; (**c**–**g**) electrochemical properties of GPE-based supercapacitor [83].

The obtained carboxylated-chitosan film with excellent flexibility exhibits the highest electrolyte uptake rate of 742.0 wt% and the highest IC of 8.69 × 10^−2^ S cm^−1^. The all-solid-state and symmetric supercapacitor showed an ED of 5.2 Wh kg^−1^ at 226.6 W kg^−1^. Figure 6 illustrates the chitosan solution in its carboxylated form and the electrochemical characteristics of the supercapacitor. The CV curves at different scan rates indicated low charge transfer resistance and ideal capacitance qualities. As the scan rate increased, the CV curve became leaf-like, showing charge transfer resistance’s increasing dominance. The GCD curves at various current densities displayed symmetric triangular shapes with minimal internal resistance drop, confirming the ideal reversible capacitance characteristics of the supercapacitor. The Nyquist plots showed a double-layer capacitive behavior with bulk resistance and charge transfer resistance in different frequency regions. The SC decreased with increasing current density due to a reduced ion diffusion and an increased electrical resistance. A study carried out by Sundriyal and co-workers presented an all-solid-state symmetrical supercapacitor based on PVA-1M Na_2_SO_4_ GPE and manganese metal–organic framework (MOF) electrodes. The introduced device exhibited an ED of 4.3 Wh kg^−1^, PD of 171.6 W kg^−1,^ and SC of 64.5 F g^−1^ [84]. Lin et al. [85] manufactured and used polyacrylamide–LiClO_4_–2-Oxazolidinone, LiClO_4_–2-Oxazolidinone (LO), and PVA–LiClO_4_–2-Oxazolidinone (PVA–LO) electrolytes to synthesize an MnO_2_-based, symmetric, and flexible supercapacitor. The supercapacitor featuring the PVA–LO GPE demonstrated superior mechanical and electrochemical performances (e.g., ED of 97.3 Wh kg^−1^ at 1200 W kg^−1^) [85]. Alternatively, a novel supercapacitor was reported on the basis of the heteroatom (N and S)-enriched AC and a LiCl/PVA-based GPE. The device prepared in that study showed an SC of 119 F g^−1^ and CR of 98% after 10,000 cycles [86]. Moreover, a supercapacitor based on 1,4-butanediol diglycidyl ether incorporating a cross-linked polyacrylamide (PAMBG) modified with cobalt (II) sulfate (PAMBGCo_X_) as a GPE electrolyte and a carbon electrode was synthesized. Excellent characteristics were observed such as an SC of 130 F g^−1^ [87]. In another study, Co was incorporated into poly(acrylic acid) (PAA) to prepare a novel GPE. PAA-Co_X_ (X: 3, 5, 7, and 10) was utilized in supercapacitor structures (quasi-solid-state devices) with carbon-based electrodes.

**Figure 7:**
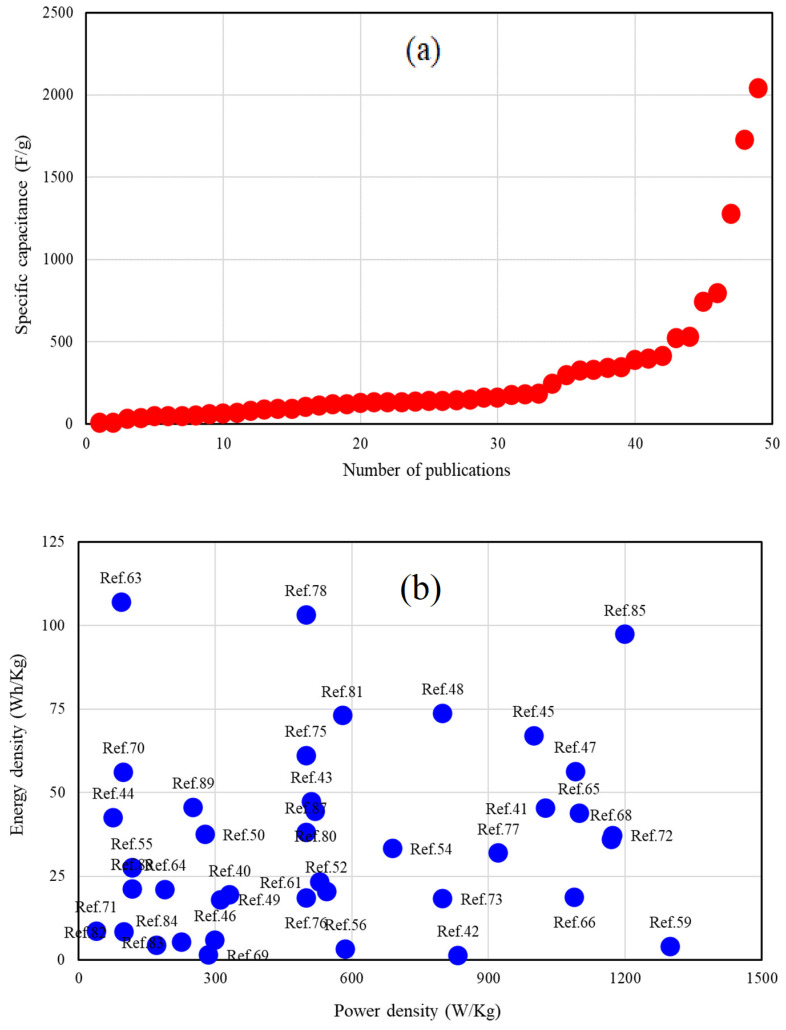
(**a**) Comparison of the specific capacitance and (**b**) Ragone plot of the GPE-based supercapacitors from Table 1, Table 2 and Table 3 [40,41,42,43,44,45,46,47,48,49,50,51,52,53,54,55,56,57,58,59,60,61,62,63,64,65,66,67,68,69,70,71,72,73,74,75,76,77,78,79,80,81,82,83,84,85,86,87].

**Figure 8:**
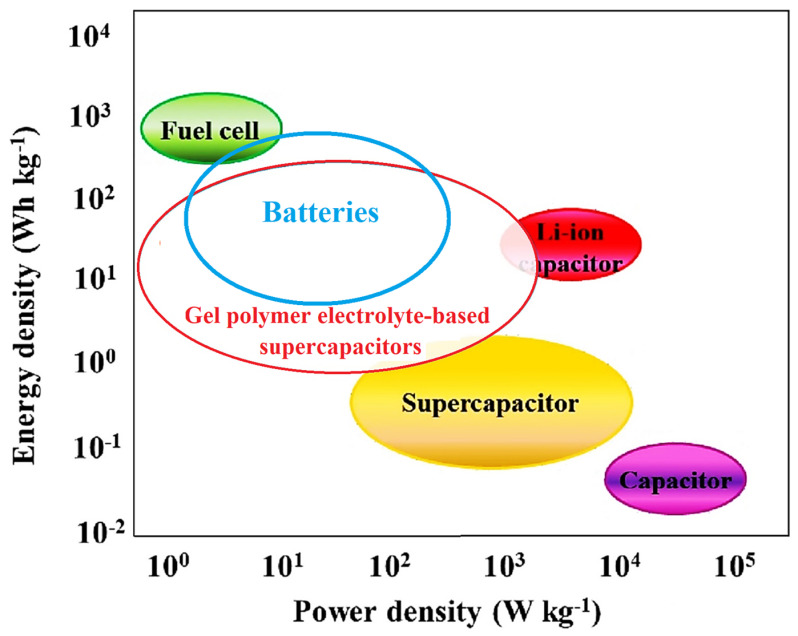
A Ragone plot of the comparison of GPE-based supercapacitors, batteries, and fuel cells.

The device revealed a superior CR of 90% after 10,000 cycles [88]. As reported by Shar et al. [89] a supercapacitor utilizing a PAA-Mo_5_ electrolyte demonstrated a significantly higher specific capacitance, approximately 15 times greater than that of a pure PAA device. Figure 7 presents a comparative analysis of the SC and Ragone plots for the GPEs-based supercapacitors. The curves indicate that the SC for the majority of supercapacitors (GPEs-based ones) remains below 1000 F g^−1^, while the ED approaches approximately 125 Wh kg^−1^, and the PD is recorded to be less than 1500 W kg^−1^. Comparing GPE-based supercapacitors with batteries can provide valuable insights into their unique advantages, limitations, and potential applications. Figure 8 shows a Ragone plot of GPs-based supercapacitors, batteries, and fuel cells. GPE-based supercapacitors and batteries serve distinct roles within the energy storage landscape. While GPE supercapacitors excel in power density, cycle life, and rapid charging capabilities, batteries provide a greater energy density and are more suitable for long-term energy storage needs.

## 5. Challenges and Future Perspectives

According to the limited research on GPEs compared to electrodes, understanding the interactions between electrodes and electrolytes is crucial for performance improvement, alongside considerations of material cost, environmental impact, and potential applications in emerging technologies. Numerous research teams are actively engaged in enhancing the advanced characteristics of GPEs, focusing on property optimization and electrochemical performance improvements. These GPEs are recognized as vital components influencing supercapacitor efficiency. The stable potential window, ionic conductivity, and both chemical and thermal stabilities of the electrolyte, along with the operational temperature range, significantly affect the performance of GPEs and their practical applications. Despite notable advancements in PVA-based GPEs, several critical challenges remain that impede technological progress and commercial viability. These challenges include low ionic conductivity, inadequate potential window values affecting energy and power density, and suboptimal salt concentrations that maximize conductivity in PVA salt-based electrolytes. To address these challenges, future research should focus on enhancing the ionic conductivity and potential window of PVA-based GPEs to boost the energy and power density of supercapacitors. This can be achieved through the incorporation of suitable modifiers and additives, as well as conducting in-depth studies on the optimization of conducting salts in SPEs, alongside theoretical and experimental investigations to deepen our understanding of these materials [90]. IL polymer electrolytes exhibit a wide range of applications beyond their role as solid-state electrolytes, including their utilization in lithium-ion batteries, fuel cells, and electrochemical sensors, thereby enhancing overall system performance. The incorporation of IL polymer electrolytes increased stability and also boosted energy storage performance in supercapacitors, making them advantageous for electric vehicles and renewable energy systems. Despite their benefits, the development of IL polymer electrolytes for supercapacitors encounters challenges, such as the need to balance a high ionic conductivity with the thick viscosity of ionic liquids, which can hinder ion mobility, particularly at lower temperatures. Additionally, the high costs and complexities associated with the synthesis of ionic liquids pose barriers to commercial viability, necessitating the exploration of more economical production methods while ensuring mechanical and electrochemical stability for advanced applications [91].

## 6. Conclusions

Today, the demand for solid-state supercapacitors with high power density and rate performances for use in soft electronics has shown an increasing trend. Solid-state electrolytes are favored for their unique advantages such as improved safety and no leakage. GPEs are preferred for energy storage due to their high IC and from a safety standpoint. GPEs typically consist of a hydrophile polymer matrix (e.g., PVA and PANI) and a conductive IL phase. Solid-state supercapacitors utilizing GPEs respond effectively to the demands of contemporary electronic applications, highlighting the importance of safety, stability, and performance. The distinctive characteristics of gel polymer electrolytes render them a highly viable option for energy storage in flexible electronic devices. This review compares the electrochemical performances of PVA/H_2_SO_4_, PVA/KOH, IL blend polymers, and different GPEs for utilization in supercapacitors. The SC for GPEs-based supercapacitors was generally found to be under 1000 F g^−1^, with an ED reaching around 125 Wh kg^−1^ and a PD below 1500 W kg^−1^.

## Figures and Tables

**Table 1:** Comparison between various supercapacitors containing PVA-based electrolytes.

Electrolyte	SC (F g^−1^)	ED (Wh kg^−1^)	PD (W kg^−1^)	Ref.
PVA-H_2_SO_4_	139.4	19.4	331.8	[40]
PVA-H_2_SO_4_	344.2	45.25	1025.64	[41]
PVA-H_2_SO_4_	45.86	1.27	833.3	[42]
PVA-H_2_SO_4_	412.7	47.3	512	[43]
PVA-H_2_SO_4_-[Co(en)_3_] Cl_3_·3H_2_O	522	42.49	76.5	[44]
PVA-H_2_SO_4_-H_3_BO_3_	134	67	1000	[45]
PVA-H_3_PO_4_	69.2	5.9	300	[46]
PVA-KOH	112.1	56.2	1091.5	[47]
PVA-KOH	1727.9	73.7	800	[48]
PVA-KOH	160	18	312	[49]
PVA-KOH	389.7	37.46	278	[50]
PVA-KOH	2041	27.8	3400	[51]
PVA/HSS-S	52	20.40	545	[52]
PVA-KOH/urea/LiClO_4_	186	125	2200	[53]
PVA-KOH/hydroquinone	326.53	33.15	689.58	[54]
PVA/glycerol/boric acid/LiNO_3_	396	27.41	118	[55]
PVA/polyethylene oxide/CH_3_COONa/Na_2_SO_4_	93.768	3.25	586.166	[56]
PVA/polyethylene glycol	105	14.8	5000	[57]

**Table 2:** Comparison of supercapacitors on the basis of IL-based electrolytes.

Electrolyte	SC (F g^−1^)	ED (Wh kg^−1^)	PD (W kg^−1^)	Ref.
ILs/BCs	90	4	1300	[59]
ILs/PVA/KI/EC/EMIBF_4_	160.6	180.67	375	[60]
ILs/Comb copolymer	37.3	23.2	530	[61]
ILs/Biopolymer	140	23.13	3600	[62]
ILs/EMIMBF_4_	180	107	94	[63]
ILs/EMITFSI/PVdF-HFP	146	21	190	[64]
ILs/SPSU/hBN	90.4	43.8	1100	[65]
ILs/SPSU/SiO_2_	134.1	18.6	1089	[66]
ILs/PVA/Li_2_SO_4_	136	10.6	3400	[67]
ILs/b-PE	150	36	1170	[68]
PPy+PIL2/PIL1/PPy+PIL2	7	1.39	286	[69]
ILs/SSPE	178	56	99	[70]
ILs/GPE	296	8.6	38.9	[71]
ILs/PIBMA-co-PEGMA	48.6	37.12	1174	[72]

**Table 3:** Comparison of various reported supercapacitors based on GPEs.

Electrolyte	SC (F g^−1^)	ED (Wh kg^−1^)	PD (W kg^−1^)	Ref.
Alkaline GPE	741	18.3	800	[73]
Li-ion GPE	6.7	12.4	6800	[74]
LiBF_4_-IL GPE	80	61	500	[75]
Cellulose GPE	133	18.5	500	[76]
Cellulose GPE	31.7	31.8	922.22	[77]
PVPA/Ni(NO_3_)_2_ GPE	793	103.1	500	[78]
PVPA/Mo GPE	1276	180.2	500	[79]
PAMPS GPE	244	44.4	520	[80]
PAMPS/Mo_3_ GPE	530	73	580	[81]
LiCl GPE	60	8.3	99.8	[82]
Chitosan GPE	45.9	5.2	226.6	[83]
PVA-Na_2_SO_4_ GPE	64.5	4.3	171.6	[84]
PVA-LiClO_4_ GPE	121.6	97.3	1200	[85]
PVA-LiCl GPE	119	21.48	14000	[86]
PAMBGCo_X_ GPE	130	38	500	[87]
PAA-Co_X_ GPE	341.33	21.25	117.69	[88]
PAA-Mo_5_ GPE	327.50	45.49	251.92	[89]

## Data Availability

The original contributions presented in this study are included in the article. Further inquiries can be directed to the corresponding author.
